# Supplementary material for: The African ape-like foot of Ardipithecus ramidus and its implications for the origin of bipedalism
Source: eLife. 2019 Apr 30;8:e44433. doi: 10.7554/eLife.44433 (PMC6491036; doi:10.7554/eLife.44433)
Supplement: Supplementary file 1. [file elife-44433-supp1.docx]

| Supplementary Table 1. Phylogenetic generalized least squares (PGLS) regression analysis | | | | | | | | | |
| --- | --- | --- | --- | --- | --- | --- | --- | --- | --- |
| trait | coefficient | s.e. | *T* | *p*-value | logLik | AIC | λ | *p*(λ = 0) | *p*(λ = 1) |
| **MT1** |  |  |  |  | 39.62 | -75.24 | 1 | < 0.001 | 1 |
| intercept | 2.979 | 0.123 | 24.249 | < 0.001 |  |  |  |  |  |
| logBM | 0.234 | 0.028 | 8.412 | < 0.001 |  |  |  |  |  |
| **MT5** |  |  |  |  | 36.81 | -69.62 | 0.956 | < 0.001 | 0.024 |
| intercept | 3.234 | 0.112 | 29.641 | < 0.001 |  |  |  |  |  |
| logBM | 0.232 | 0.027 | 8.59 | < 0.001 |  |  |  |  |  |
| **PP4** |  |  |  |  | 19.04 | -34.09 | 0.988 | < 0.001 | 0.105 |
| intercept | 2.9 | 0.183 | 15.85 | < 0.001 |  |  |  |  |  |
| logBM | 0.213 | 0.042 | 5.014 | < 0.001 |  |  |  |  |  |
| **Talar trochlea** | |  |  |  | 50.04 | -96.08 | 0 | 1 | < 0.001 |
| intercept | 1.982 | 0.026 | 76.896 | < 0.001 |  |  |  |  |  |
| logBM | 0.335 | 0.01 | 33.885 | < 0.001 |  |  |  |  |  |
| **Talar neck** | |  |  |  | 33.42 | -62.83 | 0.574 | 0.002 | < 0.001 |
| intercept | 1.439 | 0.072 | 19.958 | < 0.001 |  |  |  |  |  |
| logBM | 0.289 | 0.022 | 13.377 | < 0.001 |  |  |  |  |  |
| **Cuboid** |  |  |  |  | 42.27 | -80.55 | 0 | 1 | < 0.001 |
| intercept | 1.915 | 0.031 | 62.522 | < 0.001 |  |  |  |  |  |
| logBM | 0.271 | 0.012 | 23.022 | < 0.001 |  |  |  |  |  |
