# Supplementary material for: The African ape-like foot of Ardipithecus ramidus and its implications for the origin of bipedalism
Source: eLife. 2019 Apr 30;8:e44433. doi: 10.7554/eLife.44433 (PMC6491036; doi:10.7554/eLife.44433)
Supplement: Supplementary file 2. [file elife-44433-supp2.docx]

| Supplementary Table 2. Performance of alternative models for the evolution of the anthropoid foot | | | | | | | |
| --- | --- | --- | --- | --- | --- | --- | --- |
|  | BM | OU.1 | OU.2 | OU.3 | OU.4 | OU.5 | SURFACE |
| -2logL | -134.55 | -143.07 | -201.89 | -214.8 | -243.25 | -269.19 | -320.25 |
| aic | -116.55 | -113.07 | -159.89 | -166.8 | -189.25 | -209.19 | -242.25 |
| aic.c | -115.11 | -109.04 | -151.71 | -155.89 | -175.12 | -191.31 | -209.4 |
| sic | -90.4 | -69.49 | -98.88 | -97.07 | -110.81 | -122.03 | -128.94 |
| dof | 9 | 15 | 21 | 24 | 27 | 30 | 39 |
